# Label-free, ultra-low detection limit DNA biosensor using high quality optical microcavity functionalized by DNA tetrahedral nanostructure probes

**DOI:** 10.1515/nanoph-2023-0238

**Published:** 2023-06-20

**Authors:** Hongdan Wan, Shuai Zhang, Yu Gu, Jinxin Xiong, Ji Xu, Cheng Wan, Jie Chao

**Affiliations:** Nanjing University of Posts and Telecommunications, #9 Wenyuan Road, Nanjing, 210000, China; Suzhou Yikon Medical Laboratory Company Ltd., Xukang Medical·Yikon Genomics, Suzhou 215000, China; Jiangsu Key Laboratory for Biosensors, Institute of Advanced Materials, National Synergetic Innovation Center for Advanced Materials, Nanjing, China

**Keywords:** DNA tetrahedral nanostructure probes, label-free, limit of detection, single-nucleotide mismatch recognition, surface functionalization, whispering gallery mode

## Abstract

This study proposes and demonstrates a novel label-free DNA biosensor using high quality optical microcavity functionalized by 3D DNA nanostructure probes. To achieve ultra-low limit DNA biosensing, optical sensing interface of the hollow-core, thin wall-thickness microcavity was functionalized by self-assembled DNA tetrahedral nanostructure (DTN) probes with size of 17 bp and length of ∼5.8 nm. High efficiency binding of the DTN probes with the optical sensor interface and the target ssDNA are achieved. Whispering gallery mode (WGM) spectra with high-*Q* factor of >10^7^ are excited and traced for DNA detection inside the microfluidic channel of the microcavity, with a small sample volume of nL. Incorporation of nanoscale DTN probes onto surface of the optical microcavity makes it an effective way for increasing efficient probe density and eliminating entanglement between DNA probes, thus ∼1000 times lower detection limit is achieved as compared to using 1D ssDNA probes. Due to its desirable merits of label-free, ultra-low LoD, real time and compact size, the proposed DNA biosensor has broad application prospects in bioengineering and medical diagnosis.

## 1 Introduction

The risk of virus spreading of the coronavirus-19 (COVID-19) pandemic had made it a global threat to public health [1, 2]. There was urgent need for highly specific and sensitive diagnostic measures to identify infected people and monitor the propagation of virus-pandemic [3–5]. Multiple methods had been reported to detect coronavirus. As for the antibody detection method, the accuracy is less than satisfactory and can only be detected after the immune system responds [6]. Deoxyribonucleic acid (DNA) is the carrier of genetic information, and it is distinct in any living organism, virus, or pathogen [7, 8]. Therefore, through their specific nucleic acid sequences, the DNA biosensor can be able to discriminate different organisms and diagnose various diseases and human pathogens [9–12]. Polymerase chain reaction (PCR) technology combined with fluorescence is the current gold standard for nucleic acid detection [13]. However, due to the labeling process, the false negative is relatively high, moreover, the time cost and detection limit should be further reduced [14, 15]. There still need efforts to develop label-free, real-time DNA detection technique with low limit of detection (LoD).

Recently, micro-nano optical fiber biosensors have attracted considerable research interests in terms of biological sensing, due to their ability for label-free and real-time detection [16, 17]. Guan et al. presented a sensitive bio-probe to *in situ* detect unlabeled single-stranded DNA targets based on optical microfiber taper interferometer coated by a high ordered pore arrays conjugated polymer, the sensor exhibits the lowest detection ability of 10^−10^ M or even lower [18]. Duan et al. proposed and implemented a thin-core fiber-based double-S tapered fiber (TF) sensor for label-free DNA detection, with a LoD of <1 nM [19]. But for most micro-nano fiber biosensors, the LoD cannot be further reduced since the interaction length is limited by the single-pass of the optical fiber device [20]. Optical biosensor based on whispering gallery mode (WGM) microcavity has advantages of strong interaction and long interaction length between the optical field and the analyte for the presence of high quality (*Q*) factor [21–24]. In particular, hollow microcavities have distinguished features including perfect combination of detection and transmission of liquid samples with compact size and small amounts [25–28]. However, current optical DNA sensor is based on base complementary pairing and hybridization, using single stranded DNA (ssDNA) molecules as detection probe [29]. The density, orientation and entanglement between ssDNA probes heavily affect their recognition process, the existing of electrostatic repulsion force, nonspecific adsorption force as well as the base accumulation force between the ssDNA probes deteriorate DNA hybridization efficiency and result in difficulties in reduction of LoD [30–33].

Two methods are proposed to improve biosensors’ LoD and sensitivity. The first one is to strengthen the interaction between light field and matter, based on: (1) 2D materials (such as graphene oxide (GO), black phosphorus (BP), etc.): Wang et al. demonstrated the ultrasensitive detection of complementary DNA (cDNA) by employing a fiber optic biosensor functionalized with a nano-interface of BP, the sensor exhibits an ultrahigh sensitivity, with LoD as low as 0.24 pM [34]. (2) Surface plasmon resonance technology: Huang et al. demonstrated an optical microfiber integrated with Cu_2−*x*_S nanoplates supported by GO, which achieved ultra-high sensitivity and differential single base mismatch of miRNAs at ultra-low concentrations (as low as 10 aM) in serum by stimulating local surface plasmon resonance [35]. (3) Interface WGM modes: Xiao et al. reported the implementation of the interface modes for ultrasensitive sensing in a microbubble resonator, achieving a LoD of 0.3 pg/cm^2^ [36]. The second method is to improve molecular hybridization efficiency: Fan et al. reported self-assembled DNA nanostructures for engineering electrochemical DNA sensors’ interface, achieving LoD of 1 fM, with decreased hybridization time and increased hybridization efficiency [37]. Thus, engineering biosensing interface with nanostructure probes can be expected to solve the difficulties of improve performance of current optical DNA sensors [38, 39].

This paper, to the best of our knowledge, is the first report of optical DNA biosensor using a high *Q*, hollow-core microbottle cavity (HC-MBC) functionalized by 3D DNA tetrahedral nanostructure (DTN) probes for ultra-low limit DNA detection. Based on thermal controlled surface functionalization of the optical sensor interface, self-assembled DTN probes with optimized length of <10 nm are anchored at the inner surface of the thin-wall thickness HC-MBC with high efficiency and stability. Whispering gallery mode (WGM) spectra with high-*Q* factor of >10^7^ are excited and traced for DNA detection inside the microfluidic channel of the microcavity, with an nL-level sample volume. With reduced non-specific adsorption and entanglement of DNA probes, high efficiency DNA hybridization is achieved. Thus, ∼1000 times lower LoD of DNA detection is achieved as compared to using 1D ssDNA probes. Single-nucleotide mismatch recognition is also achieved by WGM spectral detection. Benefiting from its advantages of label-free, ultra-low LoD and volume, high specific sensing of DNA molecules is achieved, which has high research significance in various biosensing fields, such as gene diagnosis, kinship judgment, and environmental monitoring etc.

## 2 Sensor fabrication and analysis

### 2.1 Principle and analysis

**Figure 1:**
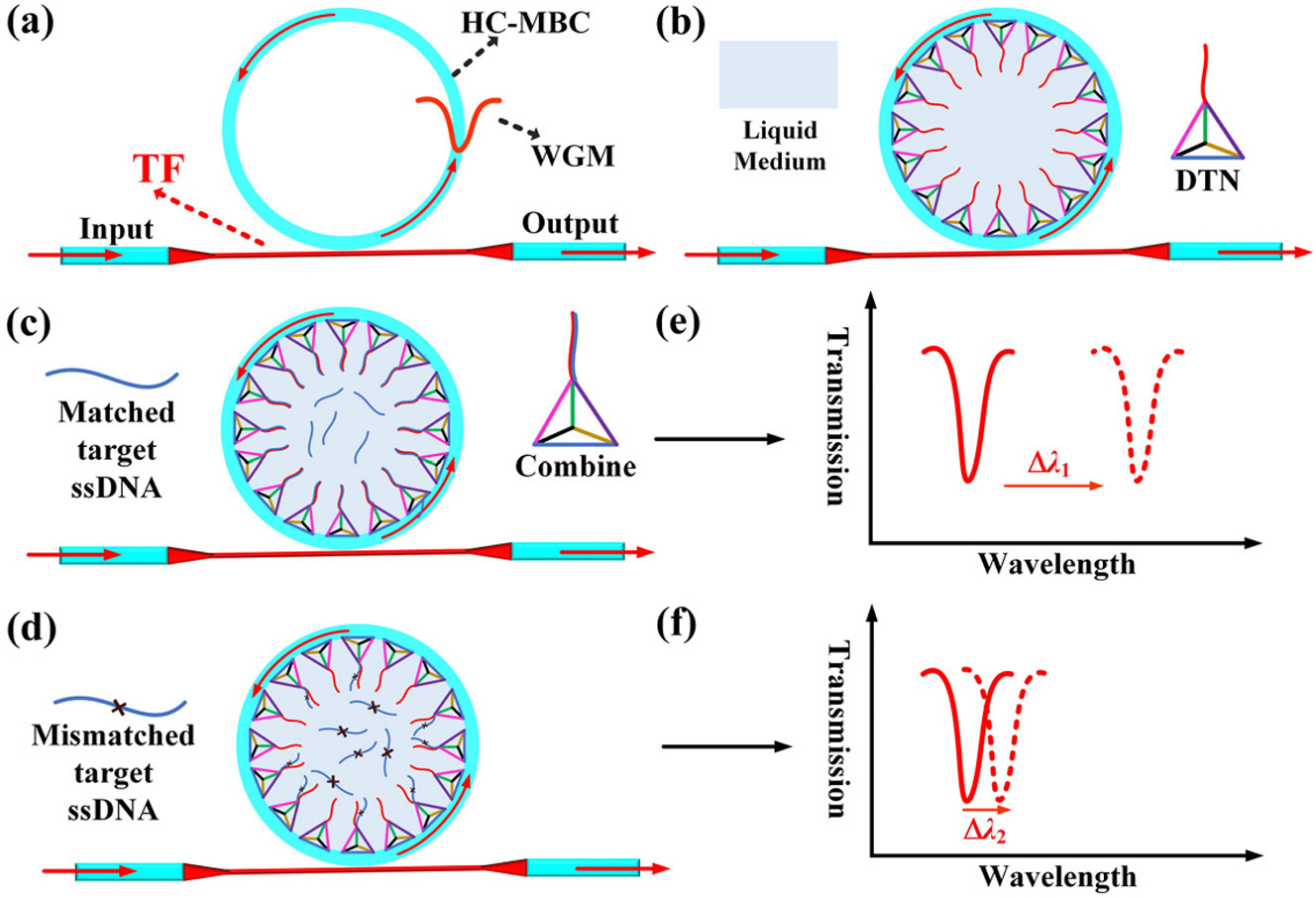
Schematic diagram of the DNA sensing principle: (a) the coupling of TF and HC-MBC; (b) DTN probe is bound onto the surface of the microcavity; DNA hybridization between: DTN probes and matched target ssDNA (c) and mismatched target ssDNA (d); WGM Spectral shift caused by matched target ssDNA (e) and mismatched target ssDNA (f).

Figure 1 shows the bio-physical sensing principle of the proposed DNA biosensor. Evanescent light generated by the TF is coupled with the HC-MBC, total internal reflection occurs in the thin-wall of the microcavity to excite the WGM oscillation (Figure 1(a)). Then, DTN probes are bound onto the inner-wall surface of the microcavity, each DTN carries a DNA probe at one vertex (Figure 1(b)). Hybridization between the probe molecules and target molecules results in variation of the refractive index (RI) and thickness of the (inner) wall surface of the microcavity (Figure 1(b)), the light propogation is thus changed and can be measured by tracing of WGM wavelength shift in real time. In Figure 1(e), Δ*λ*_1_ is the wavelength shift caused by hybridization of matched target ssDNA and DTN probes. However, for mismatched target ssDNA, less or no bonding occurs in Figure 1(f), resulting in a negligible WGM spectral shift of Δ*λ*_2_, Δ*λ*_1_ > Δ*λ*_2_. WGM wavelength shift is decided by the highly specific Watson–Crick base pairing, namely, the hybridization efficiency of well-defined 3D DNA nanostructures on the biosensing interface of the sensor.

### 2.2 Sensor fabrication and experimental setups

**Figure 2:**
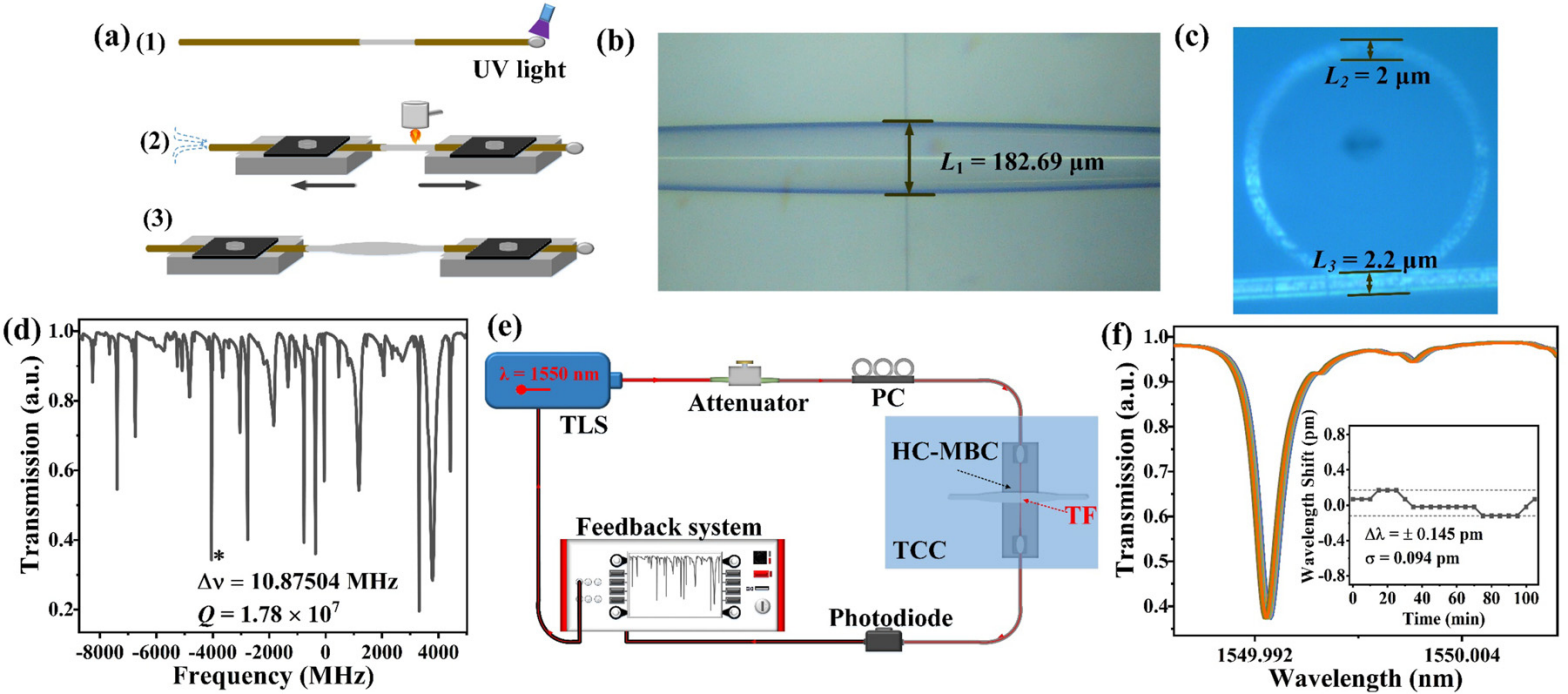
Sensor fabrication and characterization: (a) HC-MBC fabrication process; (b) microscope image of the TF coupled HC-MBC; (c) cross-sectional microscope image; (d) measured WGM spectrum with *Q* factor of ∼10^7^; (e) experimental setup of the sensor system; and (f) measured WGM spectral stability of the sensor.

The HC-MBC is fabricated by fuse-and-blow technique with a fused silica capillary, as proposed in our previous work [40]. The wall thickness of the microcavity is reduced to enhance the interaction between light and matter, as well as the sensitivity of the microcavity. The fabrication process of the HC-MBC is shown in Figure 2(a)(1), one end of the SiO_2_ capillary is sealed with ultraviolet glue. Then, as shown in Figure 2(a)(2, 3), the other end of the capillary is connected to a syringe. The coating layer of the capillary is removed and one section of the capillary is heated, softened and then stretched. Meanwhile, air pressure is controlled inside the capillary to form an HC-MBC. The geometric size of the HC-MBC and the tapered fiber (TF) is shown in Figure 2(b) and (c) characterized by following parameters: the outer diameter *L*_1_ = 182.69 μm, wall thickness *L*_2_ = 2 μm, TF diameter *L*_3_ = 2.2 μm. Figure 2(d) shows the experimentally measured WGM spectra excited by high-precision coupling of the HC-MBC to the TF. The *Q* factor can be calculated by *Q* = *λ*/Δ*λ* (*λ* is the resonance wavelength of the WGM and Δ*λ* is the 3 dB linewidth of the resonance wavelength). According to the experimental results, the *Q* factor calculated near the wavelength of 1550 nm is ∼1.78 × 10^7^.

The experimental setup is illustrated in Figure 2(e), consisting of a tunable laser source (TLS, center wavelength near 1550 nm, linewidth of ≤5 KHz, and tuning range of 35 GHz), an attenuator, a polarization controller (PC), the coupling unit, a photodetector (PD), and the feedback system. The coupling unit consists of the HC-MBC and the TF. The coupling position of the HC-MBC and the TF is adjusted precisely by 5D mechanical alignment stages. The coupling unit is placed in a constant temperature environment, using a thermal controlled container (TCC). Figure 2(f) shows the measured stability of the sensor. It can be seen that the wavelength drift of the WGM is within ±0.145 pm during 2 h, which may be still influenced by surrounding environment (air flow, vibration, etc.).

## 3 Materials and methods

### 3.1 Chemicals and materials

Piranha solution (3:1 mixture of concentrated sulfuric acid and 30 % hydrogen peroxide) and 0.1 %w/v poly-L-lysine (PLL) solution is purchased from Sigma-Aldrich company. The oligonucleotides used in this experiment were purchased from Sangon Biotech Co., Ltd (Shanghai, China). The specific information of the oligonucleotides is shown in Table 1.

**Table 1:** Sequences of the oligonucleotides.

Oligonucleotides	5′ End modification	Sequences (5′-3′)	3′ End modification
Tetra-A	None	CATCTTGCCTAAAAAAAAAAACATTCCTAAGTCTGAAACATTACAGCTTGCTACACGA	None
		GAAGAGCCGCCATAGTA	
Tetra-B	NH_2_–C_6_–	TATCACCAGGCAGTTGACAGTGTAGCAAGCTGTAATAGATGCGAGGGTCCAATAC	None
Tetra-C	NH_2_–C_6_–	TCAACTGCCTGGTGATAAAACGACACTACGTGGGAATCTACTATGGCGGCTCTTC	None
Tetra-D	NH_2_–C_6_–	TTCAGACTTAGGAATGTGCTTCCCACGTAGTGTCGTTTGTATTGGACCCTCGCAT	None
Probe ssDNA	None	CATCTTGCCTAAAAAAAAAA	-C_6_-NH_2_
Target ssDNA-Cy3	None	TTTTTTTTTTAGGCAAGATG	-Cy3
Target ssDNA	None	TTTTTTTTTTAGGCAAGATG	None
1-base mismatch ssDNA	None	TTTTTTTTTTATGCAAGATG	None

### 3.2 Synthesis of the DTN probes

**Figure 3:**
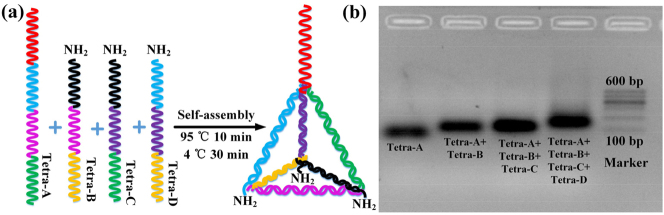
Synthesis of the DTN probes: (a) Synthesis process and (b) characterization of the synthesis efficiency.

The synthesis process of the DTN probes is shown in Figure 3(a). Tetra-A and three amino modified single strands (Tetra-B, Tetra-C, and Tetra-D) were dissolved in ultrapure water with a final concentration of 10 μM. A total 10 μL of each strand was combined with 60 μL of TM buffer (20 mM Tris, 50 mM MgCl_2_, pH 8.0). The resulting mixture was heated to 95 °C for 10 min, cooled to 4 °C in 30 s and stand for 30 min using a thermal cycler. Thus, DNA molecules are self-assembled into DTN probes with each edge contains 17 base pairs and anchored at the sensor interface. Each base pair was separated by 0.34 nm in a double helix, the edge length of the DTN probe is thus calculated to be ∼5.8 nm, and the specific morphology of the DTN probes can be characterized by atomic force microscopy (AFM). However, it is not possible to characterize structure (size and shape) of DTN probes on the surface of our hollow-core microcavity [37]. Assuming that DTN probes are closely anchored at the inner surface of HC-MBC, the corresponding density of DTN probes per square centimeter is ∼2.9 × 10^12^ cm^−2^ [37]. The synthesis efficiency of the DTN probes is characterized by gel electrophoresis, as shown in Figure 3(b), which shows that the DTNs can be self-assembled, with high yields of >95 %.

### 3.3 Surface functionalization and characterization

**Figure 4:**
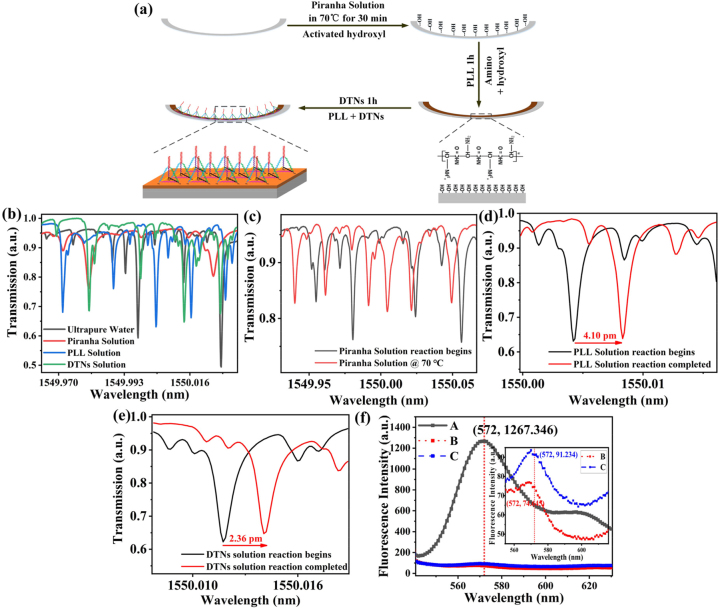
Surface functionalization and characterization: (a) experimental process. (b) Measured WGM spectra of different solutions. (c) Piranha solution; (d) PLL solution; and (e) DTNs solution. (f) Detection of binding efficiency (A: Baseline, the relative fluorescence intensity of the Cy3-labeled target ssDNA. B: the residual solution after binding of the DTN probes and target ssDNA in HC-MBC. C: after the combination of the DTN probes onto the functionalized sensor interface.).

Thermal-treated surface functionalization method of the HC-MBC is used for anchoring the DTN probes at the inner surface of the HC-MBC. The specific process is shown in Figure 4(a). Firstly, the piranha solution is injected into the HC-MBC and stood at 70 °C for 30 min to activate the negatively charged hydroxyl groups on the surface of the HC-MBC. Then, PLL solution is injected into the HC-MBC and stood for ∼1 h. The positively charged amino group in the PLL solution will produce electrostatic binding with the negatively charged hydroxyl group in the piranha solution. In the whole process of surface functionalization, the HC-MBC is cleaned with ultrapure water for 10 min before each solution change. The measured WGM spectra during the surface functionalization procedure are shown in Figure 4(b). The WGM spectra were widen and drift in this process, due to the interaction between light and matter is different for varies solutions. As shown in Figure 4(c), due to the influence of temperature of the piranha solution (in 70 °C), the WGM spectra change significantly. For example, during the reaction process of a specified solution (PLL solution or DTNs solution), the refractive index and thickness of the microcavity surface medium change, resulting in measurable wavelength shift. As shown in Figure 4(d). The WGM spectrum red-shifts for ∼4.10 pm during the reaction process of PLL solution. After surface functionalization, the DTN probe solution is injected into the HC-MBC and stood for ∼1 h. DTN probes with amino groups were connected with amino groups in PLL to produce –NH–NH– connection so as to complete the self-assembly of the DTN probes at the functionalized sensor interface, the WGM spectrum red-shifts for ∼2.36 pm during the reaction process of DTNs solution, as shown in Figure 4(e) .

Binding efficiencies between the DTN probes with functionalized sensor interface and the target ssDNA were measured by RF-6000 plus fluorescence spectrophotometer (with excitation wavelength of 530 nm, and emission wavelength range of 540–620 nm). (1) Firstly, binding efficiency between the DTN probes and the target ssDNA inside the HC-MBC was measured: 1 μM solution of the DTN probes was injected into the HC-MBC, stood for 1 h and rinsed with ultrapure water. Then, 1 μM solution of the Cy3-labeled target ssDNA was injected into the processed HC-MBC. After 1 h of reaction, the residual liquid (unbound target ssDNA) is discharged into an EP tube. (2) Secondly, binding efficiency between the DTN probes and the functionalized sensor interface was measured: A mixed solution of the DTN probes and Cy3-labeled target ssDNA with a ratio of 1:1 was synthesized by PCR and injected into the functionalized HC-MBC. After 1 h of reaction, the residual liquid is discharged into another EP tube. Due to large amount of liquid required in the cuvette for fluorescence detection, the solutions in the two EP tubes are diluted into same volume of ∼250 μL. (3) Fluorescent spectra of three kinds of solutions (with the same volume) are measured and compared: A is the reference solution, namely the original Cy3-labeled target ssDNA. B is the residual solution after the combination of the DTN probes with the target ssDNA in the HC-MBC. C is the residual solution after the combination of the DTN probes onto the functionalized sensor interface. (DTN probe solution is mixed with the Cy3-labeled target ssDNA with ratio of 1:1). As shown in Figure 4(f), the measured relative fluorescence intensity at 572 nm is 1267.346, 74.645, and 91.234 for A, B, and C, respectively. The binding efficiency can be calculated as: (((original fluorescence intensity – residual fluorescence intensity)/original fluorescence intensity) * 100 %). Thus, the binding efficiency between the DTN probes with the surface functionalized HC-MBC and the target ssDNA are 92.8 % and 94.11 %, respectively.

**Figure 5:**
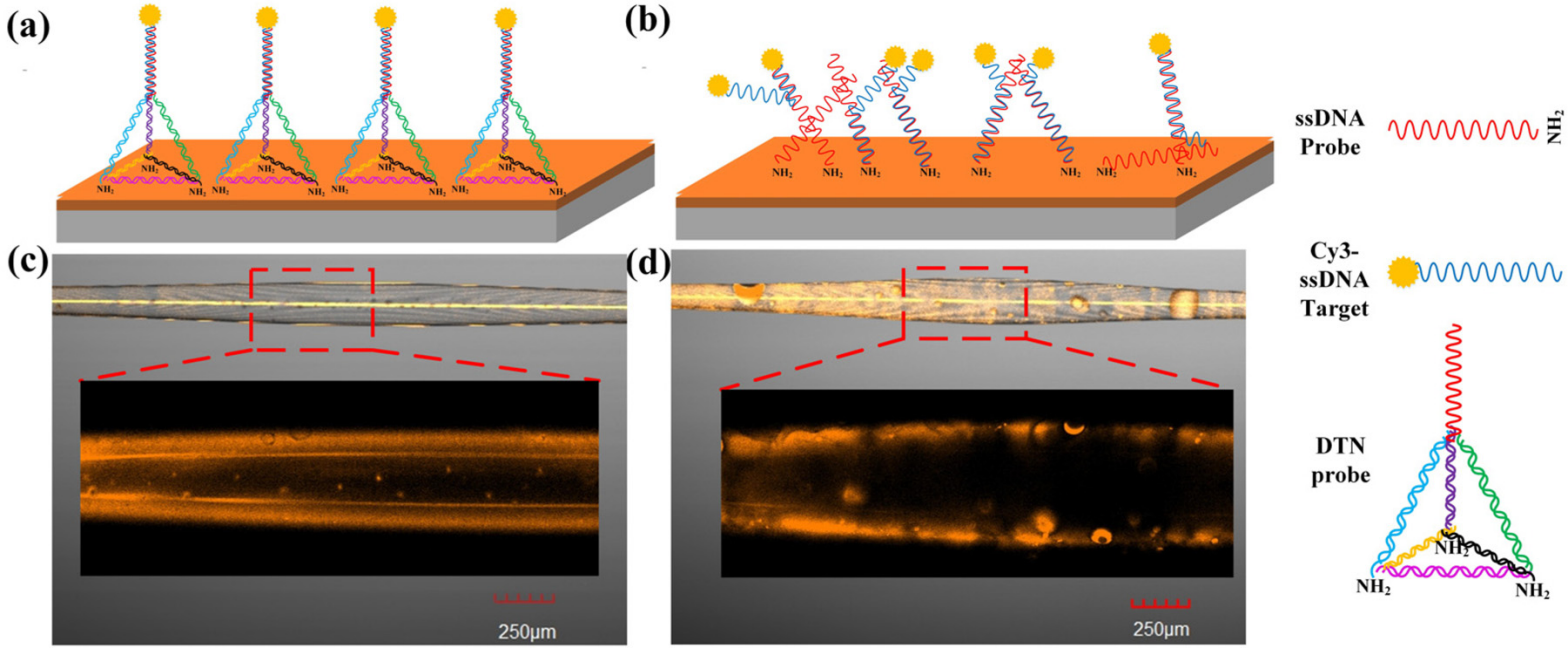
Comparison of surface functionalization regimes of the DTN probes and the ssDNA probes: (a) DTN probes anchored on the sensor interface; (b) ssDNA probes anchored on the sensor interface; fluorescence characterization of the DTN probes (c) and the ssDNA probes (d) on the thin-wall of the HC-MBC.

Surface functionalization regimes of the DTN probes and the ssDNA probes on the inner-wall of the SiO_2_ microcavity are measured and compared as shown in Figure 5(a) and (b). As compared to the ssDNA-based biosensor, using the DTN scaffold can minimize non-specific adsorption and entanglement of the DNA probes on the sensor surface (Figure 5(a)), the orientation of the DNA probes is improved in Figure 5(b), the effective number of probes is increased, thus higher efficiency for DNA hybridization can be achieved. The fluorescence microscopic images of the sensor interface are measured by a laser confocal fluorescence microscope (FV1000MPE, excitation wavelength of 517 nm, operation wavelength of 530–630 nm). 1 μM solution of the DTN probes was injected into the HC-MBC, stood for 1 h and rinsed with ultrapure water. Then, 1 μM solution of the Cy3-labeled target ssDNA was injected into the processed HC-MBC. After reacting inside the HC-MBC for 1 h. The experimental results are shown in Figure 5(c) and (d), it can be seen that the fluorescent signal distribution of the DTN probes is more uniform stronger as compared to the ssDNA probes. Thus the reactivity and accessibility of probes on the proposed sensor interface for hybridization detection is much better for using the DTN probes.

## 4 Results and discussion

### 4.1 DNA hybridization detection

**Figure 6:**
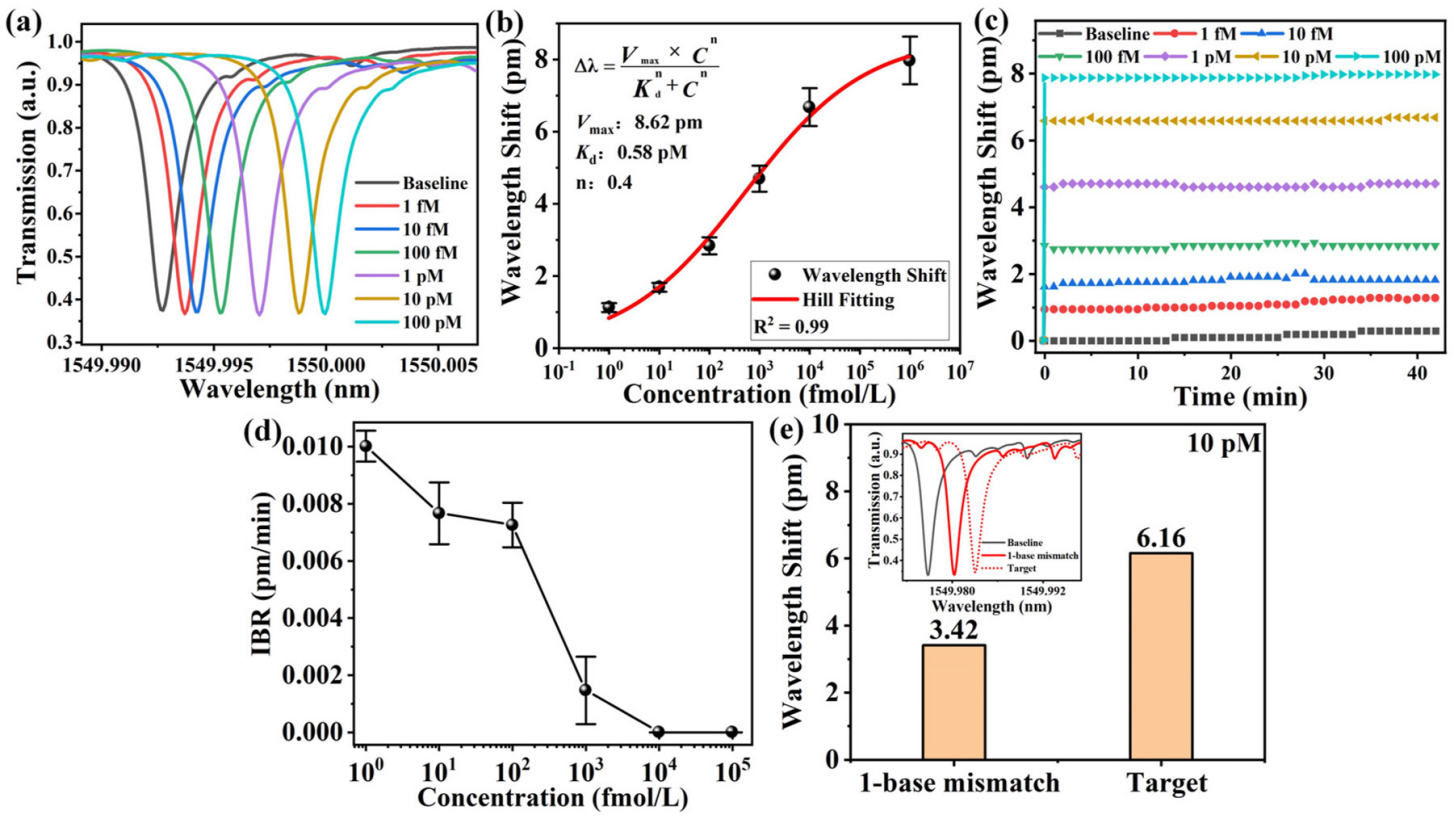
Experimentally measured DNA hybridization results: (a) WGM spectra red-shift as the target ssDNA concentration is increased. (b) Wavelength shift versus concentration of the target ssDNA. (c) Variation of WGM shift versus detection time. (d) The IBR versus target ssDNA concentration with different sequence lengths. (e) Specificity detection. The 1-base mismatched target ssDNA exhibits relatively weak signals, indicating that our sensors can sensitively distinguish single nucleotide polymorphisms.

The target ssDNA is diluted into 6 different concentrations (1 fM, 10 fM, 100 fM, 1 pM, 10 pM and 100 pM) by ultrapure water successively. Ultrapure water without target ssDNA (concentration of 0 M) is set as the reference spectra for detection. Target ssDNA solutions with increased concentration are injected into surface functionalized HC-MBC and washed by ultrapure water during each solution change. Figure 6(a) shows the measured WGM spectra are red-shift as the target ssDNA concentration is increased. Figure 6(b) shows the variation of wavelength shift versus target ssDNA’s concentration. The red line is fitted via the Hill model [41]:

(1) $$\Delta \lambda =V_{\max}\times C^{n}/\left(K_{d}^{n}+C^{n}\right)$$

Where, Δ*λ* is the relative spectral shift, *V*_max_ is the maximum possible shift observed for maximum surface coverage with target, *K*_d_ is the dissociation constant for hybridization; *C* is the bulk concentration of the target ssDNA, *n* is the Hill coefficient. Thus, the Hill fitting function is Δ*λ* = 8.62 × *C* ^0.4^/(0.58^0.4^ + *C* ^0.4^). The detection limit *x*_LOD_ is determined by blank signal (Baseline) drift *y*_blank_ and standard deviation *σ*_max_ and can be expressed as follows [42]:

(2) $$x_{\text{LOD}}=f^{-1}\left(y_{\text{blank}}+3\sigma_{\max}\right)$$

Where, *f* ^−1^ is the inverse function of the fitting function. And according to the calculation (2), the LoD of this sensor down to 260 aM, which is much lower than previous DNA sensing results using ssDNA probes 40. Figure 6(c) shows the spectral shift of the WGM spectra shift is gradually stable after a reaction time of 40 min. In order to overcome relatively long detection time and evaluate the response time, the approach involving the measurement of the initial binding rate (IBR) of the interaction was proposed in Refs [42, 43], as an effective method for determining the analyte concentration. As shown in Figure 6(c), it can be seen that the response time decreases as the target ssDNA concentration is increased. The IBR is the slope of binding in the first few minutes, as shown in Figure 6(d). When concentration increases, the detection time and the drift amount of the WGM will reduce during the detection process.

### 4.2 Sensor specificity

As to evaluate specificity of the proposed biosensor, single-nucleotide mismatch was measured. A relatively high concentration of 10 pM of the target ssDNA was chosen for single-nucleotide mismatch recognition measurement. The experimental results are shown in Figure 6(e). The inset figure demonstrates the WGM spectral shift for different target ssDNA combination with the DTN probes, namely the baseline (Ultrapure water without target ssDNA (concentration of 0 M)), the 1-base mismatched ssDNA and the target ssDNA. The experimental result shows that the wavelength shift of the WGM spectra of the 1-base mismatched and the matched target ssDNA are quite different, about 3.42 pm and 6.16 pm, respectively. Thus, the DTN-based sensors exhibited good ability to discriminate single nucleotide mismatch than the ssDNA-probe based one [40], high sensor specificity can be achieved by the proposed DNA biosensor.

In addition, the LoD of different optical DNA biosensors are listed and compared in Table 2. Thus, the proposed optical DNA biosensor has the lowest LoD.

**Table 2:** Comparison of Different optical biosensor for DNA detection.

Optical device	Surface activation	Probe type	Probe length	LoD	Ref
Microfiber Sagnac interferometer	PLL	ssDNA	26-mer	75 pM	[15]
Dual S-tapered thin-core fiber interferometer	PLL	ssDNA	25-mer	<1 nM	[18]
Microfiber-assisted Mach–Zehnder interferometer	PLL	ssDNA	21-mer	100 pM	[28]
Electrochemical	Au–S	DTN probes	20-mer	1 fM	[37]
Microfiber grating	PLL	ssDNA	26-mer	0.5 μM	[44]
Micro-capillary	PLL	ssDNA	20-mer	<2.5 μM	[45]
Long-period fiber-grating	APTES	ssDNA	15-mer	2 μM	[46]
Single-mode fiber-no-core fiber-Single-mode fiber	Gold film	ssDNA	21-mer	80 nM	[47]
HC-MBC	PLL	DTN probes	20-mer	260 aM	This work

## 5 Conclusions

In conclusion, a novel label-free DNA biosensor using high quality optical microcavity functionalized by 3D DTN probe is proposed and demonstrated. Optical sensing interface of the hollow-core, thin wall-thickness microcavity was functionalized by self-assembled DTN probes with size of 17 bp and length of ∼5.8 nm. Whispering gallery mode (WGM) spectra with high-*Q* factor of >10^7^ are excited and traced for DNA detection inside the microfluidic channel of the microcavity, with a small sample volume of nL. High efficiency binding of the DTN probes with the optical sensor interface and the target ssDNA are achieved. Incorporation of nanoscale DTN probes onto surface of the optical microcavity makes it an effective way for increasing efficient probe density and eliminating entanglement between DNA probes, thus DNA biosensing with ultra-low LoD of 260 aM is achieved, which is ∼1000 times lower as compared to using 1D ssDNA probes. Due to its desirable merits of label-free, ultra-low LoD, real time and compact size, the proposed DNA biosensor has broad application prospects in bioengineering and medical diagnosis. Specific single-nucleotide mismatch recognition is also achieved by detection of WGM spectral shift. Due to its desirable merits of label-free, ultra-low LoD, real time and compact size, the proposed DNA biosensor has broad application prospects in bioengineering and medical diagnosis.
